# Incidence of Meningitis in Patients Presenting With Febrile Seizures

**DOI:** 10.7759/cureus.11941

**Published:** 2020-12-06

**Authors:** Amr Eldardear, Faris Abdulmuti D Alhejaili, Abdullah Mohammed D Alharbi, Feras Saleh S Alrehaili, Khaled Taleb A Mohammed, Abdulmohsen Khalid A Binladin, Meshal Khaled S Aloufi

**Affiliations:** 1 Pediatric Neurology, Taibah University, Madinah, SAU; 2 Internal Medicine, Taibah University, Madinah, SAU; 3 Medicine, Taibah University, Madinah, SAU; 4 Pediatrics, Taibah University, Madinah, SAU

**Keywords:** febrile seizures, meningitis, lumber puncture

## Abstract

Introduction: Febrile seizures are very commonly encountered in the setting of the pediatric emergency department; it represents 72.2% of seizures presenting to the pediatric emergency department in Saudi Arabia and affects about 3-8% of children. Febrile seizures are usually benign and treated conservatively. This is in contrast to bacterial meningitis, which carries a fatality rate of 14.4%. Meningitis presents with seizures in 23% of cases. Differentiation between febrile seizures and meningitis is therefore of utmost importance to avoid poor outcomes. On the other hand, this may cause many patients with febrile seizures to get exposed to unnecessary invasive testing. This study aims to define the incidence of meningitis in patients with febrile seizures and the proportion of these patients who undergo invasive lumbar puncture.

Methods: This retrospective cross-sectional study was conducted at the Maternity and Children's Hospital in Madinah, Saudi Arabia. All patients presenting with febrile seizures in the period between January 2015 and June 2019 were covered. Patients' data were gathered from the hospital database and files. Descriptive analysis was performed using SPSS.

Results: A total of 1375 patients were studied, with a male-to-female ratio of 1.44:1. The median age of the sample was 24 months (interquartile range: 13 - 42). Lumbar puncture was done for 108 (7.67%) of them. Only nine patients (8.3%) had meningitis, while the other 99 (91.7 %) had no meningitis.

Conclusion: Febrile seizures are a common disease among children. The distinction between febrile seizures and meningitis is paramount to avoid poor outcomes. Bacterial meningitis is rare among patients with febrile seizures. The clinical judgement remains the cornerstone in deciding which patients should undergo invasive testing.

## Introduction

A febrile seizure is generally defined as a seizure occurring with a febrile illness. The early literature did not exclude seizures that may have been associated with an underlying intracranial cause such as meningitis [1]. The definition of the 1980 febrile seizures consensus committee excluded seizures associated with an intracranial infection or had another identifiable cause or those presenting with a history of non-febrile seizures [2]. The International League Against Epilepsy (ILAE) 1993 committee established a more comprehensive definition of febrile seizures, “An epileptic seizure occurring in childhood after the age of one month, associated with a febrile illness not caused by an infection of the CNS, without previous neonatal seizures or a previous unprovoked seizure, and not meeting criteria for other acute symptomatic seizures” [3]. Febrile seizures usually affect children between the ages of five months and six years with a prevalence of 3-8%, which makes them the most common type of seizures in childhood [4]. A recent study conducted in Arar, Saudi Arabia found that febrile seizures were the aetiology in 72.2% of all cases of seizures in the paediatric ER [5]. Clearly, febrile seizures are a common issue, but they are usually considered benign and the treatment is generally supportive, including general principles of emergency care and abortion of the seizure, although most febrile seizures resolve spontaneously before arrival to the emergency department [6]. Febrile seizures are subdivided into simple and complex, simple febrile seizures are generalized, lasting less than 15 minutes and do not recur within 24 hours. In contrast, complex febrile seizures are either focal, prolonged (>15 minutes), or seizures that recur within 24 hours [7]. Bacterial meningitis is one of the deadliest infections, affecting both adults and children, and defined as inflammation of the meninges covering the brain [8]. While the worldwide incidence is difficult to determine, the median incidence globally in children was estimated to be 34 per 100,000 child-years, with a median fatality rate of 14.4% [9,10]. Thus, early recognition and treatment are imperative to avoid poor outcomes [7]. No recent local studies were done to explore the incidence of meningitis, but one old multi-centre study in Saudi Arabia reported an incidence of only 12 in 10,000 among patients presenting to the hospital, and there was an older study in Medina, Saudi Arabia that reported an annual rate of 3.2% among hospitalized patients [11,12]. The substantial increase could be attributed to the increased reported cases of meningitis during that period due to outbreaks in Hajj and Umrah pilgrimage [13]. Also, the introduction of mandatory vaccinations played a major role in the reduction of cases. Diagnosis of meningitis can often be difficult in the absence of signs of meningism, especially in children less than two years of age [14]. Meningitis is associated with seizures in 23% of cases, and one study in Saudi Arabia found that 20% of patients with confirmed bacterial meningitis had seizures [15,16]. Another study reported an even higher figure of 28.8% seizure occurrence in meningitis patients [11]. It is extremely rare for a simple febrile seizure to be the sole manifestation in patients with bacterial meningitis [15]. Yet one older study found that 70% of practitioners perform a lumbar puncture in patients presenting with febrile seizures [17]. This, combined with the high prevalence of febrile seizures in children means that many children may get exposed to unnecessary invasive procedures [18]. In this study, we are trying to find out the incidence of meningitis among patients presenting with febrile seizures, and explore the common medical practice of performing lumbar puncture in patients with febrile seizures.

## Materials and methods

A retrospective cross-sectional study was conducted at the maternity and children’s hospital in Madinah, Saudi Arabia covering all patients who were diagnosed with or suspected of having febrile seizures in the period between January 2015 and June 2019. The patients’ data were gathered from the hospital database and analysed. The data included patient’s demographics, clinical presentation and lab results. We included patients who presented with a seizure attack and a temperature of ≥38 °C (during or just after the seizure). Any patients who had metabolic disturbances, a history of a non-febrile seizure or epilepsy syndrome, or those having major congenital or structural abnormalities were excluded. Descriptive analysis was performed using SPSS (IBM Corp., Armonk, NY).

## Results

A total of 1375 patients who presented to the hospital with febrile seizures in the period between January 2015 and June 2019 were identified, with a median age of 24 months (interquartile range: 13 - 42 months), 809 (58.8%) of the patients were males and 566 (41.2%) were females (Table 1).

**Table 1:** Descriptives of the sample.

Mean age	33.52±0.77 months
95% confidence interval	Lower border: 31.99 months
	Upper border: 35.04 months
Median age	24 months
Interquartile range	13–42 months

Out of those 1375 patients, 56 (4.1%) were younger than six months, 280 patients (20.4%) were between the ages of 6 and 12 months, and 169 (12.2%) were between 12 and 18 months, while 870 (63.3%) were older than 18 months. Of the 1375 patients, only 108 had undergone a lumbar puncture (7.85%), and the rest were assumed to have no meningitis based on not undergoing lumbar puncture (Table 2).

**Table 2:** Age distribution of the sample.

Age	Number of patients	Percent (%)
≤6 months	56	4.1
6–12 months	280	20.4
12–18 months	169	12.2
≥18 months	870	63.3
Total	1375	100

Of the 108 patients who underwent lumbar puncture, there were 7 (13.2%) male patients who were ≤6 months, and 25 (47.2%) between the ages of 6 and 18 months, and 21 (39.6%) ≥18 months. While there were 13 (23.6%) female patients who were ≤6 months, 22 (40%) between 6 and 18 months, and 20 (36.4%) ≥18 months (Table 3).

**Table 3:** Age and gender distribution of patients who received lumbar puncture.

Age	Male, N (%)	Female, N (%)
≤6 months	7 (13.2%)	13 (23.6%)
6–18 months	25 (47.2%)	22 (40%)
≥18 months	21 (39.6%)	20 (36.4%)
Total	53 (100%)	55 (100%)

In total, a lumbar puncture was performed on 53 males and 55 females with a median age of 13 months (interquartile range: 8-24 months). The ones who tested positive for meningitis were five males (9.4%) and four females (7.3%). Overall, only nine patients (8.3%; 95% confidence interval: 0.18-5.39 months) had meningitis, while the other 99 (91.7 %; 95% confidence interval: 1.64-2.57 months) did not (Table 4).

**Table 4:** Descriptives of (A) patients who received lumbar puncture and (B) patients diagnosed with meningitis.

(A) Patients who received lumbar puncture	
Mean age	2.16±0.23 months
95% Confidence interval	Lower border: 1.7 months
	Upper border: 2.6 months
Median age	13 months
Interquartile range	8–24 months
(B) Patients diagnosed with meningitis	
Mean age	33±1.13 months
95% Confidence interval	Lower border: 0.18 months
	Upper border: 5.39 months
Median age	15 months
Interquartile range	8–48 months

**Figure 1:**
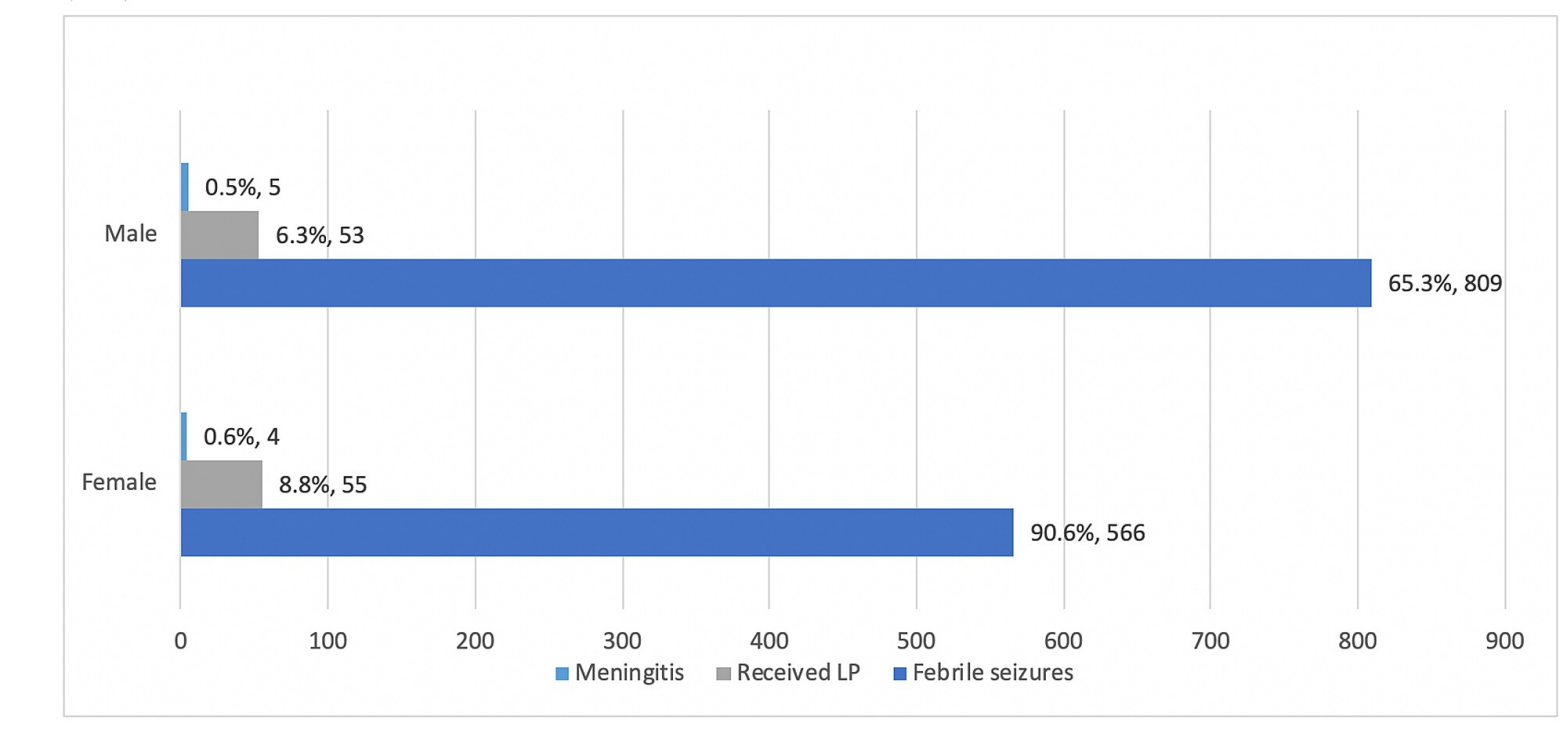
Number and percentage of patients with febrile seizures, those who received lumbar puncture and those diagnosed with meningitis.

Of the five males who had meningitis, two (40%) of them aged between 6 and 12 months, while the other three (60%) were ≥18 months. While in females, one (25%) was ≤6 months, and one (25%) was between 6 and 12 months, while the other two (50%) were between 12 and 18 months (Figure 1 and Table 5).

**Table 5:** Age and gender distribution in patients who were diagnosed with meningitis.

Age	Male, N (%)	Female, N (%)
≤6 months	0 (%)	1 (25%)
6–12 months	2 (40%)	1 (25%)
12–18 months	0 (0%)	2 (50%)
≥18 months	3 (60%)	0 (0%)
Total	5 (100%)	4 (100%)

## Discussion

Out of the total number of patients presenting with febrile seizures, males constituted a higher proportion when compared to females, with a male-to-female ratio of 1.44:1. This is consistent with the figures in a recently reported meta-analysis of 1.6:1 male-to-female ratio [19]. The results of the study clearly show that meningitis is not common among patients presenting with febrile seizures, with an overall incidence of 63.9 in 10,000 (0.64%). This is, however, significantly higher (p<0.05) than the previously reported national incidence of 12 in 10,000, but it is up for debate whether this is enough difference to merit a higher index of suspicion of meningitis in patients with febrile seizures. In the study done by Casasoprana et al., their reported incidence of bacterial meningitis was much higher than this study, at 1.9% among patients with febrile seizures [20]. In the studies done by Fletcher and Sharieff and Kimia et al. where discrimination between simple and complex febrile seizures was done and only patients with complex febrile seizures were included, the reported incidence among the total population in those studies was 0.5% and 0.57%, respectively [21,22]. Other studies in developing countries showed an even higher incidence of meningitis among patients with febrile seizures. A study in Ghana done by Owusu-Ofori et al. found an incidence of 3.13% and Tavasoli et al. reported an incidence of 1.07% in Iran [23,24].

The distinction between the two types of febrile seizures is important, as reported by Casasoprana et al., there was a significant difference between simple and complex febrile seizures regarding the risk of having a serious central nervous system infection (bacterial meningitis or encephalitis) in patients with complex febrile seizures when compared to simple febrile seizures (14% versus 0%) [20]. A recent meta-analysis reported less disparity between the two types of febrile seizures (0.2% versus 0.6%) [25]. We could not explore this issue due to deficiency of reporting in the patient’s files, as there was no discrimination between simple or complex febrile seizures in patient’s reports.

There was no significant difference in gender distribution of patients with bacterial meningitis (P>0.05). Similar results were reported by Owusu-Ofori et al. who found 19 cases of bacterial meningitis among 608 patients with febrile seizures; 9 were males and 11 were females [23]. In the study done by Tavasoli et al., they also reported a similar result, 19(4.5%) cases of meningitis among 681 patients with febrile seizure. Eleven patients (58%) were males and nine patients (42%) were females [24].

Lumbar puncture was performed on 108 out of 1408 patients (7.67%); this rate is definitely lower than previously reported rates in other recent studies. Casasoprana et al. reported that 40% of patients with febrile seizures were subjected to lumbar puncture in Toulouse, France [20]. Kimia et al. reported a higher rate of 65%, and Fletcher et al. reported a similar rate of 70.5% [21,22]. It is important to note, however, that both Kimia et al. and Fletcher et al. only included patients with complex febrile seizures in their studies, while the study done by Casasoprana et al. -similar to our study- did not make a distinction between the two types of seizures [20-22]. One limitation of our figure is that we could not account for the frequency of patients who were offered lumbar puncture but refused; this is an important note to stress as a recent multi-center study done in Saudi Arabia reported a 44.3% refusal rate by parents who were offered lumbar puncture for their children for suspicion of meningitis [26].

Vaccination must have played a very important role in reducing the incidence of meningitis among Saudi children in general, including patients with febrile seizures, which could explain the low incidence of bacterial meningitis in our sample. Studies done in the late 1980s and early 1990s showed that *Haemophilus influenza* type b was the causative organism in the majority of cases with bacterial meningitis compared to *Neisseria meningitidis* which was more common in other parts of the world [27-29]. This was attributed to the compulsory vaccination program introduced in that era, in which immunization against *N. meningitidis* was part of it [29]. Vaccination against *H. influenzae* type b was integrated into the vaccination program after those studies had concluded. The recent epidemiological studies of bacterial meningitis support the notion that the Saudi immunization program was highly effective in reducing the incidence of bacterial meningitis [11].

## Conclusions

Febrile seizures are one of the most common presentations in the pediatric emergency department. While the disease is usually benign in nature, the distinction between it and bacterial meningitis is paramount to avoid poor outcomes associated with bacterial meningitis. Bacterial meningitis is rare among patients with febrile seizures and it is not often necessary to expose this population to redundant invasive testing. Clinical judgement and balance between risk and reward remain the cornerstone in deciding which patients are more likely to benefit from the lumbar puncture.
